# Perceived parenting styles and chronic pain course in mid-life in the general population

**DOI:** 10.1192/j.eurpsy.2026.11480

**Published:** 2026-07-31

**Authors:** I. Rouch, J.-M. Dorey, M.-P. Strippoli, B. Laurent, C. Van de Leur, S. Ranjbar, A. von Gunten, M. Preisig

**Affiliations:** 1 Saint Etienne hospital, Saint Etienne; 2 U1219, INSERM, Bordeaux; 3 old age psychiatry, CH le Vinatier, Lyon, France; 4 CHUV, Lausanne, Switzerland; 5 U1028, INSERM, Lyon, France; 6 CEPP, CHUV, Lausanne, Switzerland

## Abstract

**Introduction:**

Insecure attachment is a possible risk factor for later pain characteristics in clinical populations with chronic pain (CP). However, few studies have addressed this question in the general population.

**Objectives:**

This study assessed the associations of perceived parenting styles with incident, persistent and remitted CP during a 5-year follow-up in middle-aged community-dwellers as well as the potentially moderating effect of sex on these associations.

**Methods:**

Data stemmed from the two first follow-up (FU1 and FU2) evaluations of CoLaus|PsyCoLaus, a prospective cohort study conducted in the general population of Lausanne, Switzerland. Parental bonding was assessed using the Parental Bonding Instrument (PBI). The 1,739 participants included in the analysis were divided into four groups according to their CP status at FU1 and FU2: (1) incident CP, (2) persistent CP, (3) remitted CP, and (4) CP free (reference group). The associations between PBI sub-scores and CP status were assessed with multinomial logistic regressions controlling for various confounding factors including lifetime major depressive disorder (MDD) and neuroticism.

**Results:**

Sex and PBI subscores did not interact regarding CP status during follow-up. The only signficant association was that between higher denial of autonomy by the father and a higher risk of reporting persistent CP at follow-up (OR: 1.42 (1.05 ;1.91) p=0.02).

**Conclusions:**

Our results suggest that the paternal bonding style of psychological denial of autonomy may contribute to the subsequent risk of CP in mid-life. Prospective studies following adolescents into adulthood are necessary to determine the direction of this association.

**Disclosure of Interest:**

None Declared

